# Targeting staphylococcal enterotoxin B binding to CD28 as a new strategy for dampening superantigen-mediated intestinal epithelial barrier dysfunctions

**DOI:** 10.3389/fimmu.2024.1365074

**Published:** 2024-03-06

**Authors:** Carola Amormino, Emanuela Russo, Valentina Tedeschi, Maria Teresa Fiorillo, Alessandro Paiardini, Francesco Spallotta, Laura Rosanò, Loretta Tuosto, Martina Kunkl

**Affiliations:** ^1^ Department of Biology and Biotechnologies “Charles Darwin”, Sapienza University of Rome, Rome, Italy; ^2^ Department of Biochemical Sciences “A. Rossi Fanelli”, Sapienza University of Rome, Rome, Italy; ^3^ Laboratory affiliated to Instituto Pasteur Italia-Fondazione Cenci Bolognetti, Rome, Italy; ^4^ Institute of Molecular Biology and Pathology, CNR, Rome, Italy; ^5^ Neuroimmunology Unit, IRCCS Santa Lucia Foundation, Rome, Italy

**Keywords:** staphylococcal enterotoxin B (SEB), superantigen (s), T cells, CD28, inflammation, intestinal epithelial barrier dysfunctions

## Abstract

*Staphylococcus aureus* is a gram-positive bacterium that may cause intestinal inflammation by secreting enterotoxins, which commonly cause food-poisoning and gastrointestinal injuries. Staphylococcal enterotoxin B (SEB) acts as a superantigen (SAg) by binding in a bivalent manner the T-cell receptor (TCR) and the costimulatory receptor CD28, thus stimulating T cells to produce large amounts of inflammatory cytokines, which may affect intestinal epithelial barrier integrity and functions. However, the role of T cell-mediated SEB inflammatory activity remains unknown. Here we show that inflammatory cytokines produced by T cells following SEB stimulation induce dysfunctions in Caco-2 intestinal epithelial cells by promoting actin cytoskeleton remodelling and epithelial cell-cell junction down-regulation. We also found that SEB-activated inflammatory T cells promote the up-regulation of epithelial-mesenchymal transition transcription factors (EMT-TFs) in a nuclear factor-κB (NF-κB)- and STAT3-dependent manner. Finally, by using a structure-based design approach, we identified a SEB mimetic peptide (pSEB_116-132_) that, by blocking the binding of SEB to CD28, dampens inflammatory-mediated dysregulation of intestinal epithelial barrier.

## Introduction

*Staphylococcus aureus* (*S. aureus*) is a commensal opportunistic gram-positive bacterium that colonizes around 20-30% of people, but it can become pathogenic for the presence of virulence and immune evasion factors, causing different diseases ranging from mild skin infections to toxic shock syndrome (TSS) and multi-organ failure (1–4). The bacterium is a component of the nasopharynx microbiota that may colonize secondary sites including skin, throat, armpit, groin and intestine (2), where it can survive thanks to factors and enzymes that allow its adherence to the epithelial surfaces and proliferation (5). The persistent proliferation and survival of *S. aureus* at the site of infection often lead to dysbiosis with serious life-threatening conditions, including chronic inflammation (6). For example, *S. aureus* infections may cause chronic intestinal inflammation, such as staphylococcal enteritis, an inflammatory disorder common in infants and older people (7), which can also occur in healthy adults exposed to methicillin-resistant *S. aureus* (MRSA) (8, 9). The enteropathic effects and intestinal epithelial dysfunctions observed in staphylococcal enteritis have been also related to the activity of staphylococcal enterotoxins (SEs) (10), the most common cause of food-poisoning and gastrointestinal injuries (11, 12).

​SEs may act as superantigens (SAgs) and activate a large population of T lymphocytes to produce inflammatory cytokines (13). In particular, SEB directly activate T cells bearing TCRVβ3, 12, 13.2, 14, 17 and 20 (14), widely expressed in the human population (15), and CD28 costimulatory molecules either in the presence or absence of the major histocompatibility complex class II (MHC-II) or B7.1/CD80 and B7.2/CD86 molecules on antigen presenting cells (APCs) (16–23). Indeed, we recently highlighted that both SEB and SEA can directly bind the TCR and CD28, activating inflammatory signalling in the absence of APCs expressing B7 and/or MHC-II (23). Staphylococcal SAg-induced T cell activation leads to a massive uncontrolled production of inflammatory cytokines such as TNF-α, IL-6, IFN-γ, IL-17A and IL-22 (23) that may affect the integrity and functions of the intestinal mucosa.

The intestinal mucosa is composed of three main interconnected layers that provide a physical barrier. These include the mucus layer, which is the body’s first-line defence, the epithelial and the inner layers that form the mucosal immune system (24–26). The epithelial layer consists of epithelial cells interconnected and linked each other by cell-cell adhesion molecules, which provide the epithelium with the integrity and cellular activity required for its specific functions. The apical tight junctions (TJs) comprise integral transmembrane proteins, including occludins and claudins, linked to the cytosolic zona occludens (ZO) proteins (27). TJs create a permeable barrier that generally limits paracellular transport and maintains the polarity of epithelial cells by interacting with filamentous actin (F-actin) and myosin (28). In addition, cell-cell adhesion is also maintained by adherent junctions such as E-cadherin that form a complex with β- and p120-catenin determining its connection to the actin cytoskeleton (29, 30). Alterations in intestinal permeability as well as in TJs and E-cadherin organization and functions have been observed following the exposure of the intestinal epithelium to staphylococcal SEs including SEB (31–33). However, it remains unknown whether the enterotoxic activities are mediated by T-cell dependent SAg inflammatory activity of SEs (34).

Here, we show that inflammatory cytokines elicited by SEB-mediated stimulation of T cells are able to dysregulate the intestinal epithelial barrier function of Caco-2 through the downregulation of cell-cell adherent junctions and the up-regulation of transcription factors associated to epithelial mesenchymal transition (EMT-TFs) in a nuclear factor-κB (NF-κB)- and STAT3-dependent manner. By using computational design, we also identified a novel SEB mimetic peptide that, by blocking SEB/CD28 interaction, dampens inflammatory-mediated dysregulation of the intestinal epithelial barrier.

## Materials and methods

### Cells, antibodies and reagents

Human primary T cells or CD4^+^ T cells were isolated from peripheral blood mononuclear cells (PBMC) of buffy coats of anonymous healthy donors (HD) (Policlinico Umberto I, Sapienza University of Rome, Italy) by using a EasySep™ isolation kits (#17951 and #17952, STEMCELL Technology, CAN). HD signed the informed consent and the Ethic Committee of Policlinico Umberto I approved the procedure (ethical code N., 1061bis/2019, 13/09/2019). T cells were cultured in RPMI-1640 containing 5% human serum (Euroclone, UK), L-glutamine, penicillin and streptomycin. The sorted population was > 95% pure, as evidenced by flow cytometry.

Caco-2 epithelial cell line (ATCC, Maryland, USA) was cultured in DMEM supplemented with 10% FBS (Euroclone, UK), L-glutamine, penicillin and streptomycin.

The following antibodies were used: rabbit anti-human vimentin (#5741), rabbit anti-human NF-κB/p65 (#8242), rabbit anti-human phosphoTyr705-STAT3 (#9145) were from Cell Signalling Technologies (MA, USA); mouse anti-human E-cadherin (#610182), mouse anti-human N-cadherin (#610254) were from BD Biosciences (Milan, Italy); rabbit anti-human FAS (#sc-715), mouse anti-human GAPDH (#sc-47724) were from Santa Cruz Biotechnology (Texas, USA); rabbit anti-human ZO-1 (#40-2200), Alexa Fluor 594 phalloidin (#A12381), goat anti-mouse Alexa-flour 488 (#A11070), goat anti-rabbit Alexa Fluor 647 (#A21245) were from Thermo Fisher Scientific (MA, USA). Inflammatory cytokines neutralising Abs (NAbs) used were mouse anti-TNF-α (#MAB610), anti-IFN-γ (#MAB2852), anti-IL6 (#MAB206), anti-IL-17A (#MAB317) and anti-IL-22 (#MAB7822) (R&D Systems, USA). All NAbs were used at 2.5 μg ml^-1^ final concentration.

Staphylococcal Enterotoxin B (SEB, #54881) was purchased by Merck (Milan, Italy). 4′,6-diamidino-2-phenylindole dihydrochloride (DAPI, #H-1200) was from Vector Laboratories, Inc. (Burlingame, CA, USA). pSEB_116-132_ mimetic peptide (GGVTEHNGNQLDKYRSI) containing D-Ala at both termini to enhance protease resistance was purchased by BIO-FAB research (Rome, Italy). Peptide was > 95% pure and used in culture at 10 μM final concentration. PS1145 (#P6624, Merck) and S31-201 (#sc-204304, Santa Cruz Biotechnology) inhibitory drugs were used at 10 μM and 100 μm, respectively.

### Cytokine production: ELISA assays

T cells (0.5 x 10^6^ ml^-1^) were cultured in 24-well plate or 24-well plate inserts when co-cultured with Caco-2 cells (2.5 x 10^5^ ml^-1^) and stimulated for different times with 0.1 μg ml^-1^ SEB. Secretion of inflammatory cytokines (TNF-α, IL-6, IL-17A, IL-22 and IFN-γ) in culture supernatants was measured by ELISA using human TNF-α (#DY-210), IL-6 (#DY-206), IFN-γ (#DY285), IL-17A (#DY-317) and IL-22 (#DY-782) kits (R&D Systems). The assays were performed in duplicate and data analyses was performed on a Bio-Plex (Bio-Rad, Hercules, CA, USA). The sensitivity of the assays was as follow: IL-6 and IFN-γ = 9.4 pg ml^-1^, TNF-α and IL-17A = 15.6 pg ml^-1^ and IL-22 = 31.2 pg ml^-1^.

### Western blotting

Caco-2 cells (2.5 × 10^5^ ml^−1^) were co-cultured with T cells (0.5 x 10^6^ ml^−1^) in 24 trans-well plate inserts for different times at 37°C. At the end of incubation, cells were lysed for 30 min on ice in 1% Nonidet P-40 lysis buffer (0.15 M NaCl, 0.02 M Tris-HCl pH 7.5, 0.001 mM EGTA and MgCl_2_, 0.05 M NaF, 0.01 M Na_4_P_2_O_7_) containing proteases and phosphatases inhibitors (10 μg ml^-1^ leupeptin, 10 μg ml^-1^ aprotinin, 1 mM NaVO_4_, 1 mM Pefablock-SC). Proteins were resolved by SDS-PAGE and blotted onto nitrocellulose membranes. Blots were incubated with anti-E-cadherin, anti-ZO-1, anti-N-cadherin or anti-vimentin (1:1000 dilution) or anti-GAPDH (1:400 dilution) Abs, extensively washed and after incubation with horseradish peroxidase (HRP)-labelled goat anti-rabbit (#NA934V, 1:5000 dilution) or HRP-labelled goat anti-mouse (#NA931V, 1:5000 dilution) developed with the enhanced chemiluminescence’s detection system (GE Healthcare Life Sciences, Italy) and analysed by ChemiDoc XRS^+^ (Bio-Rad Laboratories, Italy). Protein levels were quantified by densitometric analysis using the ImageJ 1.50i program (NIH, USA)

### Cytotoxicity assay

Caco-2 (2.5 x 10^5^ ml^-1^) were co-cultured with T cells (0.5 x 10^6^ ml^−1^) unstimulated or stimulated with 0.1 μg ml^-1^ SEB in 24-well inserts plates. After 72 hours, Caco-2 cells were stained with 10 μg ml-1 propidium iodide (PI) and the percentage of PI-positive cells was quantified by flow cytometry (FACScalibur, BD Biosciences, Mountain View, CA).

### Confocal microscopy

Caco-2 cells (2.5 × 10^5^ ml^−1^) were co-cultured with T cells (0.5 x 10^6^ ml^−1^) in 24 trans-well plate inserts for different times. After fixing (2% paraformaldehyde) and permeabilization (0.1% saponin), E-cadherin and ZO-1 staining was performed by using mouse anti-human E-cadherin (1:100 dilution) followed by goat anti-mouse Alexa flour 488 (1:100 dilution), and rabbit anti-human ZO-1 (followed by goat anti-rabbit Alexa flour 647 (1:100 dilution). Alexa fluor 594 phalloidin (1:1000 dilution) was used to stain filamentous actin (F-actin) and DAPI was used to stain nuclei. Observations were performed with a Nikon Eclipse Ti2 confocal microscope (63X oil objective). The same acquisition settings were used to process the Z stack images by NIS Elements AR 5.30 software (Nikon Europe B.V.).

### Microscopy analysis of RelA/NF-κB and pSTAT3 nuclear translocations

Caco-2 cells were treated as indicated and the nuclear translocation of RelA/NF-κB and pSTAT3 was analysed by using anti- RelA (1:300 dilution) and anti- pSTAT3 (1:300 dilution) Abs followed by goat anti-rabbit Alexa-flour 488 (1:100 dilution). Nuclei were stained with DAPI. Images of RelA and pSTAT3 localization were obtained with a computer-controlled Nikon Eclipse 50i epifluorescence microscope and a plan achromat microscope objective 100XA/1.25Oil OFN22 WD 0.2 and QImaging QICAM Fast, 1394 Digital Camera, 12-bit-Mono (Minato, Tokyo, Japan). The fluorescence intensities of nuclear RelA and pSTAT3, overlapping with DAPI, were quantified by using Fiji ImageJ software and intensities ratio were calculated for each cell. At least one hundred cells were examined quantitatively for each condition in three independent experiments.

### Real-time PCR

Total RNA was extracted using Trizol (Thermo Fisher Scientific) from 2.5 × 10^5^ Caco-2 cells, reverse-transcribed into cDNA, and real-time PCR was performed by Luna^®^ Universal qPCR Master Mix (NEB, M3003E). The relative quantification was performed using the comparative cycle threshold (C_T_) method. Specific primer/probe sets (**Table 1**) to quantify the expression level of ZO-1, CDH1, SNAIL-1, TWIST-1, ZEB-1 and GAPDH mRNA levels were purchased by Metabion International AG (Planegg, Germany).

**Table 1:** Primer sequences of investigated genes and promoters.

Primer	Sequence (5’-3’)
Human ZO-1gene	**Forward** GGAGAGGTGTTCCGTGTTGT **Reverse** GAGCGGACAAATCCTCTCTG
Human CDH1 gene	**Forward** TACACTGCCCAGGAGCCAGA **Reverse** TGGCACCAGTGTCCGGATTA
Human SNAIL-1 gene	**Forward** TAGGCCCTGGCTGCTACAAG **Reverse** CACGCCTGGCACTGGTACTT
Human TWIST-1 gene	**Forward** TTCTCGGTCTGGAGGATGGA **Reverse** CCACGCCCTGTTTCTTTGAAT
Human ZEB-1 gene	**Forward** GGGAGGAGCAGTGAAAGAGA **Reverse** TTTCTTGCCCTTCCTTTCTG
Human GAPDH gene	**Forward** GGAAGGTGAAGGTCGGAGTC **Reverse** TCCTGGAAGATGGTGATGGG
Human SNAIL-1 promoter (NF-κB3-STAT3) -984 bp to -849 bp	**Forward** CATCCCTGGAAGCTGCTCTC **Reverse** CGTTAAGAGGCGGGTCACCT
Human SNAIL-1 promoter (NF-κB2) -484 bp to -383 bp	**Forward** TTTCCCTCGTCAATGCCACGC **Reverse** ACACCTGACCTTCCGACGC
Human TWIST-1 promoter (NF-κB-STAT3) -176 bp to -2 bp	**Forward** GGCCAGGTCGTTTTTGAATGG **Reverse** TCCGTGCAGGCGGAAAGTTT
Human ZEB-1 promoter (STAT3) -1056 bp to -928 bp	**Forward** ATCACATCTGTCAGCCGATGC **Reverse** TAGAACCGTGGGATCCTAGGT
Human ZEB-1 promoter (NF-κB) -599 bp to -482 bp	**Forward** CCCCAAACCTGCCCTTCC **Reverse** GCCTGCCTGCTTCCTGGA

### Chromatin immunoprecipitation (ChIP)

Caco-2 cells were cultured for 8 h with the supernatant of primary T cells unstimulated or stimulated for 72 h with 0.1 μg ml^-1^ SEB. ChIP assays were performed as previously described (35). Briefly, after fixing in 1% formaldehyde, Caco-2 cells were lysed and chromatin sheared by sonication. Lysates were incubated at 4°C with anti-RelA (1:100 dilution), anti pSTAT3 (1:100 dilution), or anti-FAS (1:100 dilution) Abs as control, and immune complexes were collected with sperm–saturated Protein-A Sepharose beads. After protein-DNA cross-links reversion, DNA was extracted by phenol-chloroform and analysed by real-time PCR with Luna^®^ Universal qPCR Master Mix. Specific primer/probe sets for the human SNAIL-1, TWIST- and ZEB-1 promoters were used (**Table 1**). Specific enrichment was calculated by using the C_T_: 2^(controlChIP−controlInput)^/2^(specificChIP−specificInput)^ as previously described (36).

### Structural modelling of the bivalent interaction of SEB with the TCR and CD28

Structures and complexes modelling were performed as previously described (23). The following experimentally determined structures was used to this purpose: SEB (PDB: 1SEB) (37); CD28 extracellular domain (PDB: 1YJD) (38); TCR (TRAV22/TRBV19) (PDB:4C56) (39). Briefly, full-length proteins were modelled with AlphaFold2 (40) and docked with ClusPro 2.0 (41), HADDOCK (42), and MultiLZerD (43). Protein sequence manipulations, superpositions and modelling were carried out using PyMod 3.0 (44).

### Statistical analysis

The sample size was chosen based on previous studies to ensure adequate power. Statistical analysis (mean and standard error of mean, SEM) was performed through Prism 8.0 (GraphPad Software, San Diego, CA), by using Student’s *t* test or one-way ANOVA with the Fisher’s LSD test for multiple comparisons. For all tests, P values < 0.05 were considered significant.

## Results

### SEB-activated T cells produce inflammatory cytokines and alter F-actin dynamics in Caco-2 cells

**Figure 1:**
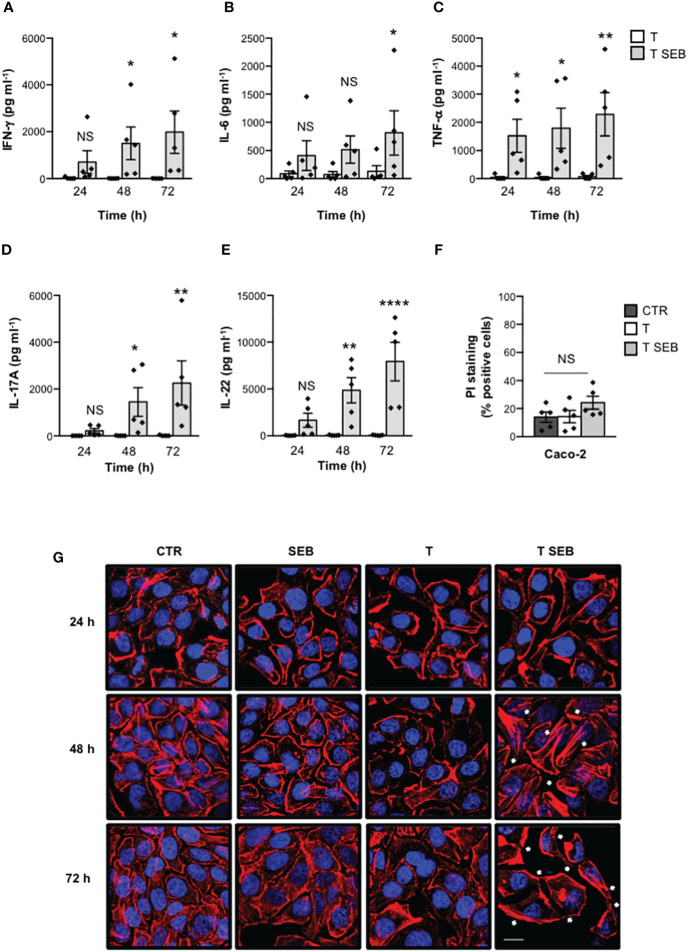
SEB-stimulated T cells secrete inflammatory cytokines and induce alterations of both permeability and morphology in Caco-2 cells. **(A–E)** Human T cells isolated from the peripheral blood of healthy donors (HD) were unstimulated (T) or stimulated for different times with 0.1 μg ml^-1^ SEB (T SEB). IFN-γ **(A)**, IL-6 **(B)**, TNF-α **(C)**, IL-17A **(D)** and IL-22 **(E)** levels in culture supernatant were measured by ELISA. Data show the mean ± SEM (standard error of the mean) of different HD (n = 5). Statistical significance was calculated by one-way ANOVA. Mean (pg ml^-1^) ± SEM values (n = 5): IFN-γ **(A)** 24 h, T = 20.74 ± 17.93, T SEB = 708.3 ± 486.1; 48 h, T = 5.29 ± 3.29, T SEB = 1505 ± 698; 72 h, T = 4.34 ± 2.52, T SEB = 1983 ± 903.5; IL-6 **(B)** 24 h, T = 86.96 ± 49.57, T SEB = 408.7 ± 267.0; 48 h, T = 74.8 ± 51.27, T SEB = 516.2 ± 243.7; 72 h, T = 129.4 ± 100.3, T SEB = 812.7 ± 394.7; TNF-α **(C)** 24 h, T = 38.55 ± 35.91, T SEB = 1522 ± 587.7; 48 h, T = 35.81 ± 33.88, T SEB = 1792 ± 712.7; 72 h, T = 69.56 ± 41.9, T SEB = 2285 ± 767.3; IL-17A **(D)** 24 h, T = 0 ± 0, T SEB = 218.8 ± 96.34; 48 h, T = 3.51 ± 3.51, T SEB = 1448 ± 609.3; 72 h, T = 9.07 ± 9.07, T SEB = 2252 ± 944.3; IL-22 **(E)** 24h, T = 28.82 ± 10.46, T SEB = 1665 ± 758; 48 h, T = 48.88 ± 14.99, T SEB = 4852 ± 1363; 72 h, T = 55.07 ± 14.38, T SEB = 7929 ± 2055. **(F)** Caco-2 cells were cultured in 24 trans-well plates for 72 h with medium alone (CTR) or T cells (T), or T cells stimulated with 0.1 μg ml^-1^ SEB (T SEB). Cell death was analysed by flow cytometry by quantifying the ability of cells to incorporate propidium iodide (PI). Data show the mean % PI positive cells ± SEM of different HD (n = 5). Statistical significance was calculated by one-way ANOVA. Mean (%) ± SEM values (n = 5); CTR = 14.06 ± 3.65, T = 14.38 ± 4.38, T SEB = 24.22 ± 4.55. (*) p < 0.05, (**) p < 0.01, (****) p < 0.0001, NS = not significant. **(G)** Caco-2 cells were cultured in 24 trans-well plates for the indicated times with medium alone (CTR) or T cells (T), or T cells stimulated with 0.1 μg ml^-1^ SEB (T SEB) for 24, 48 or 72 h. After fixing and permeabilization, F-actin was stained with 594-conjugated phalloidin (red) and analysed by confocal microscopy. Nucleus was stained with DAPI (blue). The white arrows indicate the tight parallel bundles or stress-like fibers. Scale bar = 20 μm.

We have recently demonstrated that staphylococcal SAgs may trigger inflammatory cytokine production by binding the TCR and CD28 in the absence of APCs expressing MHC-II- and/or B7 (23). Consistently with these data and with the dose-response production of inflammatory cytokines (**Supplementary Figure S1A**), stimulation of T cells with 0.1 μg ml^-1^ SEB induced a significant secretion of IFN-γ (**Figure 1A**), IL-6 (**Figure 1B**), TNF-α (**Figure 1C**), IL-17A (**Figure 1D**) and IL-22 (**Figure 1E**) after 48-72 hours (h). Since inflammatory cytokines produce by SEB-stimulated T cells have been described to alter the homeostasis and integrity of intestinal epithelial cells (45), we co-cultured SEB-activated inflammatory T cells with Caco-2 cells in trans-well plates in time-course experiments. Caco-2 is a cell line derived from a human colorectal adenocarcinoma that acquires *in vitro* the morphological and functional features of mature enterocytes of the small intestine, representing a good model of intestinal barrier (46, 47). Thereafter, both Caco-2 cell viability (**Figure 1F**) and intestinal epithelial barrier integrity (**Figure 1G**) were assessed. While no change in Caco-2 cell viability was observed (**Figure 1F**), confocal microscopy analysis revealed changes in F-actin organization in Caco-2 cells co-cultured with SEB-activated T cells (**Figure 1G**). For instance, unstimulated Caco-2 cells (CTR) displayed an extensive actin network with F-actin mainly organised into cortical bundles closely associated with cell-cell contacts. Neither SEB alone nor unstimulated T cells (T) significantly affected the steady-state of actin cytoskeleton organization as well as Caco-2 cell morphology. On the contrary, within 24 h of co-culture, Caco-2 cells exhibited para-cellular gaps, which further increased after 48-72 h (**Figure 1G**). Moreover, after 48 h of co-culture, SEB-activated T cells induced changes in F-actin dynamics in Caco-2 cells with the formation of tight parallel bundles and/or stress-like fibers (white arrows), which further increased after 72 h (**Figure 1G**).

These data suggest a potential role of inflammatory cytokines produced by SEB-stimulated T cells in altering F-actin dynamic and intestinal barrier integrity.

### The inflammatory milieu of SEB-stimulated T cells alters the integrity of the epithelial barrier by affecting the expression of cell-cell adhesion molecules and EMT-TFs

**Figure 2:**
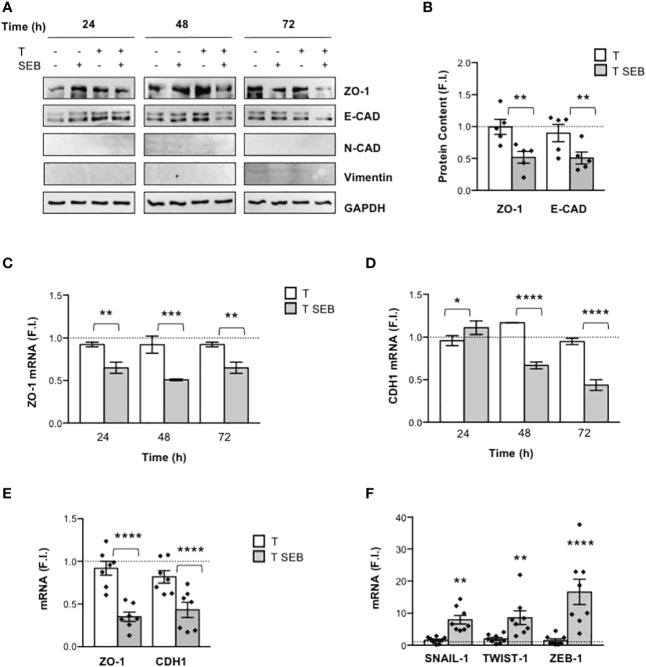
SEB-stimulated T cells induce the down-regulation of E-CAD and ZO-1, and the up-regulation of EMT-TFs in Caco-2 cells. **(A)** Representative anti-ZO-1, anti-E-cadherin (E-CAD), anti-N-cadherin (N-CAD), anti-vimentin and anti-GAPDH western blotting of Caco-2 cells cultured in 24 trans-well plates for the indicated times with medium alone or with T cells (T), or T cells stimulated with 0.1 μg ml^-1^ SEB (T SEB). **(B)** E-CAD and ZO-1 proteins levels of Caco-2 cells cultured with medium alone or with T cells from HD (n = 5) or with SEB-stimulated T cells for 72 h were quantified by densitometric analysis. Fold inductions (F.I.) over the basal level of Caco-2 cells cultured with medium alone were calculated after normalization to GAPDH levels. Data show the mean F.I. ± SEM and statistical significance was calculated by one-way ANOVA. Mean (F.I.) ± SEM values (n = 5): ZO-1, T = 0.99 ± 0.11, T SEB = 0.51 ± 0.09; E-CAD, T = 0.89 ± 0.13, T SEB = 0.50 ± 0.09. **(C, D)** Caco-2 cells were cultured in triplicates in 24 trans-well plates with medium alone or T cells (T), or SEB-stimulated T cells (T SEB) for the indicated times. ZO-1 **(C)** and E-CAD (CDH1) mRNA levels **(D)** in Caco-2 cells were measured by real-time PCR and values, normalized to GAPDH, expressed as fold inductions (F.I.) over the basal level of Caco-2 cultured with medium alone. Data show the mean F.I. ± SEM of one out of three HD. Statistical significance was calculated by one-way ANOVA. Mean (F.I.) ± SEM values (n = 2): ZO-1 **(C)** 24 h, T = 0.92 ± 0.02, T SEB = 0.64 ± 0.06; 48 h, T = 0.92 ± 0.10, T SEB = 0.50 ± 0.01; 72 h, T = 0.92 ± 0.02, T SEB = 0.64 ± 0.06; CDH1 **(D)** 24 h, T = 0.95 ± 0.05, T SEB = 1.10 ± 0.07; 48 h, T = 1.16 ± 0.002, T SEB = 0.66 ± 0.04; 72 h, T = 0.94 ± 0.03, T SEB = 0.43 ± 0.06. **(E, F)** ZO-1 and CDH1 **(E)** and the EMT-TFs SNAIL-1, TWIST-1 and ZEB-1 **(F)** mRNA levels in Caco-2 cells cultured for 72 h with medium alone or with T cells from HD unstimulated (T) or SEB-stimulated (T SEB). Values, normalized to GAPDH, were expressed as fold inductions (F.I.) over the basal level of Caco-2 cultured with medium alone. Data show the mean F.I.± SEM and statistical significance was calculated by one-way ANOVA. Mean (F.I.) ± SEM values; [**(E)**, n = 7] ZO-1, T = 0.92 ± 0.08, T SEB = 0.35 ± 0.05; CDH1, T = 0.81 ± 0.07, T SEB = 0.43 ± 0.08; [**(F)**, n = 8] SNAIL-1, T = 1.56 ± 0.36, T SEB = 7.97 ± 1.33; TWIST-1, T = 1.95 ± 0.47, T SEB = 8.59 ± 2.14; ZEB-1, T = 1.47 ± 0.54, T SEB = 16.66 ± 3.93. (*) p < 0.05, (**) p < 0.01, (***) p < 0.001, (****) p < 0.0001.

Since F-actin remodelling observed in Caco-2 cells exposed to SEB-activated inflammatory T cells resembled those occurring during epithelial-mesenchymal transition (EMT) (48, 49), we analysed the expression of E-cadherin (E-CAD) and ZO-1 (epithelial markers) as well as of N-cadherin (N-CAD) and vimentin (mesenchymal markers). The kinetic analysis pointed out that both ZO-1 and E-CAD were strongly down-regulated after 72 h of co-culture of Caco-2 cells with T cells activated with SEB (**Figures 2A, B**). On the contrary, no detectable expression of N-CAD and vimentin was observed (**Figure 2A**). The reduction of both E-CAD and ZO-1 protein content in Caco-2 cells cultured with SEB-activated T cells was also accompanied by the inhibition of ZO-1 (**Figure 2C**) and E-CAD mRNA levels (CDH1, **Figure 2D**), which started to decrease within 24 h and reached a peak of inhibition after 72 h (**Figure 2E**).

The transcriptional downregulation of ZO-1 and E-CAD observed in Caco-2 cells co-cultured with SEB-stimulated T cells prompted us to analyse the expression of the EMT-transcription factors (TFs) SNAIL, TWIST and ZEB, known epithelial marker repressors (50). Interestingly, we found a strong up-regulation of SNAIL-1, TWIST-1 and ZEB-1 expression in Caco-2 cells co-cultured for 72 h with SEB-stimulated T cells (**Figure 2F**), suggesting that these effects might be linked to E-CAD and ZO-1 downregulation. Of note, the comparison of Caco-2 cells co-cultured with total T cells or CD4^+^ T cells evidenced no significant differences in cell-cell junction downregulation and EMT-TF upregulation (**Supplementary Figure S1B**), supporting our previous data showing that most of the inflammatory cytokines produced by T cells following SEB stimulation derive from CD4^+^ T cells (23).

**Figure 3:**
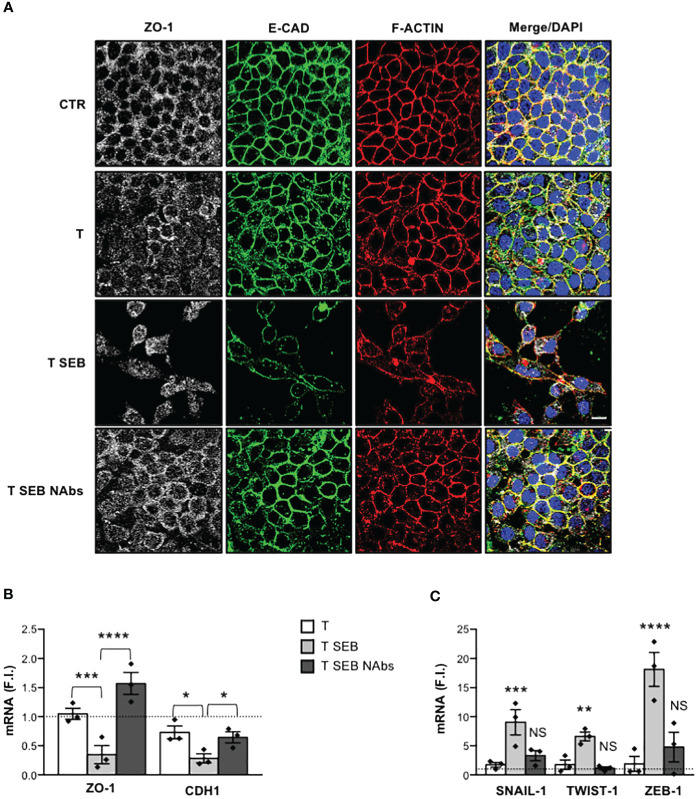
Inflammatory cytokines produced by SEB-stimulated T cells mediate Caco-2 cell dysfunctions. **(A)** Caco-2 cells were cultured in 24 trans-well plates for 72 h with medium alone (CTR) or T cells (T), or T cells stimulated with 0.1 μg ml^-1^ SEB (T SEB) in the presence of 2.5 μg ml^-1^ isotype control or 2.5 μg ml^-1^ anti-IL-6, anti-TNF-α, anti-IFN-γ, anti-IL-17A Abs neutralising Abs (NAbs). After fixing and permeabilization Caco-2 cells were stained with anti-ZO-1 followed Alexa Fluor 647 Abs (white), anti-E-CAD followed by Alexa Fluor 488 Abs (green), and F-actin was stained with Alexa Fluor 594 phalloidin (red). Nucleus was stained with DPI (blue). Scale bar = 20 μm. **(B, C)** ZO-1 and CDH1 **(B)**, and SNAIL-1, TWIST-1 and ZEB-1 **(C)** mRNA levels in Caco-2 cells cultured for 72 h with medium alone or with T cells from HD (n =3) or SEB-stimulated T cells (T SEB) in the presence of isotype control or cytokine NAbs. Values, normalized to GAPDH, were expressed as fold inductions (F.I.) over the basal level of Caco-2 cultured with medium alone. Data show the mean F.I. ± SEM and statistical significance was calculated by one-way ANOVA. Mean (F.I.) ± SEM values (n = 3); **(B)** ZO-1, T = 1.05 ± 0.09, T SEB = 0.34 ± 0.15, T SEB NAbs = 1.57 ± 0.18; CDH1, T = 0.73 ± 0.10, T SEB = 0.28 ± 0.07, T SEB NAbs = 0.65 ± 0.09; **(C)** SNAIL-1, T = 1.73 ± 0.40, T SEB = 9.04 ± 2.18, T SEB NAbs = 3.30 ± 0.87; TWIST-1, T = 1.75 ± 0.80, T SEB = 6.60 ± 0.76, T SEB NAbs = 1.13 ± 0.25; ZEB-1, T = 1.88 ± 1.30, T SEB = 18.13 ± 2.91, T SEB NAbs = 4.78 ± 2.53. (*) p < 0.05, (**) p < 0.01, (***) p < 0.001, (****) p < 0.0001. NS, not significant.

Finally, to assess whether Caco-2 cell dysfunctions were mediated by the inflammatory cytokines produced by SEB-stimulated T cells, Caco-2 cells were cultured with anti-TNF-α, anti-IFN-γ, anti-IL-6, anti-IL-17A and anti-IL-22 neutralising antibodies. Indeed, Caco-2 cells express all the receptors for these inflammatory cytokines and are susceptible to their activity (51–54). The neutralization of each cytokine did not significantly affect neither ZO-1 and CDH1 downregulation (**Supplementary Figure S2A**) nor SNAIL-1, TWIST-1 and ZEB-1 up-regulation (**Supplementary Figure S2B**) induced in Caco-2 cells by SEB-activated T cells. However, when a cocktail of all neutralising Abs (NAbs) was added in culture, we observed a significant impairment of F-actin remodelling as well as E-CAD and ZO-1 delocalization in Caco-2 cells co-cultured with SEB-stimulated T cells (**Figure 3A**). Moreover, neutralization of inflammatory cytokines also restored ZO-1 and CDH1 gene expression (**Figure 3B**) and inhibited SNAIL-1, TWIST-1 and ZEB-1 expression (**Figure 3C**) induced in Caco-2 cells by SEB-activated T cells.

Thus, inflammatory cytokines produced by SEB-stimulated T cells disrupt Caco-2 intestinal epithelial barrier and cell-cell adhesion.

### Barrier dysfunctions induced in Caco-2 cells by SEB-stimulated inflammatory T cells depend on RelA/NF-κB and STAT3

**Figure 4:**
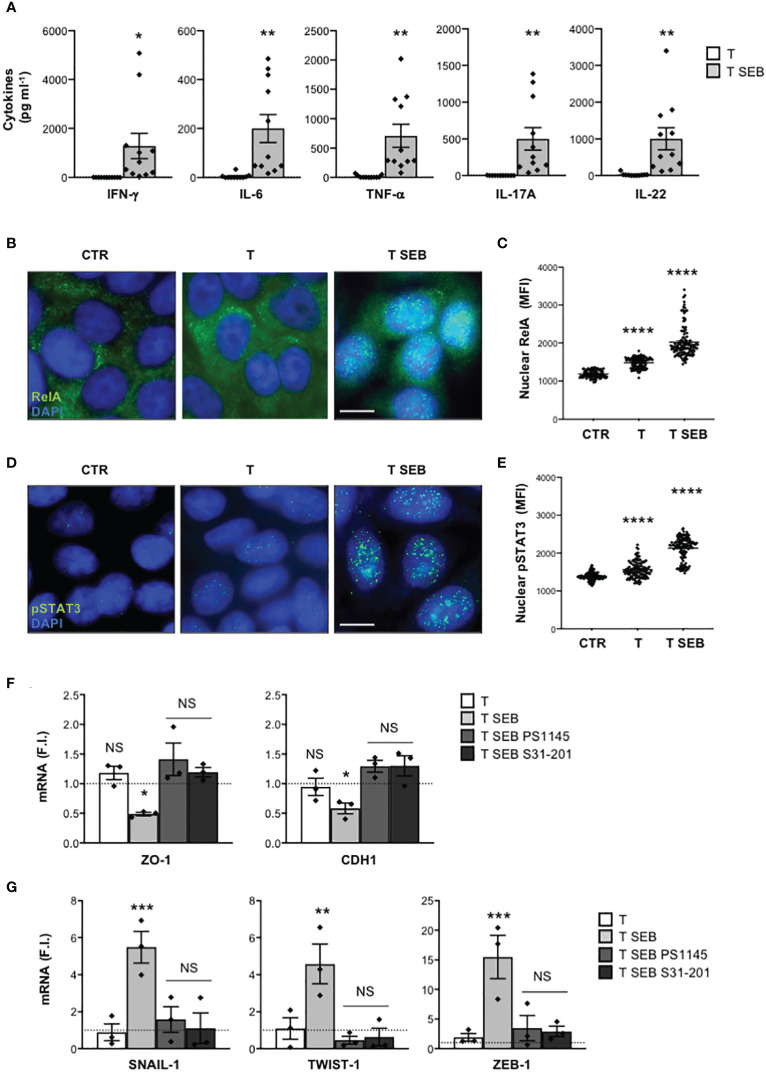
The down-regulation of adhesion molecules and the up-regulation of EMT-TFs in Caco-2 cells exposed to the inflammatory milieu of SEB-stimulated T cells depend on pSTAT3 and RelA/NF-κB TFs. **(A)** Inflammatory cytokine levels in the supernatants of peripheral T cells from HD (n = 11) stimulated with SEB for 72 h. Data show the mean ± SEM and statistical significance was calculated by Student’s t test. Mean (pg ml^-1^) ± SEM values (n = 11): IFN-γ, T = 1.71 ± 0.73, T SEB = 1289 ± 519.4; IL-6, T = 4.618 ± 3.04, T SEB = 200.2 ± 57.1; TNF-α, T = 15.54 ± 7.31, T SEB = 709.5 ± 196.6; IL-17A, T = 0.49 ± 0.49, T SEB = 500.4 ± 152.7; IL-22, T = 24.28 ± 12.12, T SEB = 1004 ± 297.8. **(B–E)** Fluorescence microscopy imaging of RelA **(B)** or pSTAT3 **(D)** nuclear translocation in Caco-2 cells cultured for 8 h with medium alone (CTR) or the culture supernatants (1:4 dilution) of T cells (T) or T cells stimulated with SEB for 72 h (T SEB). Mean fluorescence intensity (MFI) fold increase of nuclear RelA **(C)** or pSTAT3 **(E)** over unstimulated cells was calculated. Data show the mean MFI ± SEM and statistical significance was calculated by one-way ANOVA. Mean MFI ± SEM values: RelA **(C)** CTR (n = 96) = 1183 ± 9.8, T (n = 115) = 1419 ± 12.8, T SEB (n = 121) = 2024 ± 37.56; pSTAT3 **(E)**, CTR (n = 114) = 1384 ± 9.8, T (n = 120) = 1562 ± 19.39, T SEB (n = 130) = 2129 ± 26.63. **(F, G)** ZO-1 and CDH1 **(F)**, and SNAIL-1, TWIST-1 AND ZEB-1 **(G)** mRNA levels in Caco-2 cells untreated or treated with DMSO, as vehicle control, or 10 μM PS1145, or 100 μM S31-201 and cultured for 24 h with medium alone (CTR) or the culture supernatants (1:4 dilution) of T cells (T) or T cells stimulated with SEB for 72 h (T SEB). Values, normalized to GAPDH, were expressed as fold inductions (F.I.) over the basal level of Caco-2 cultured with medium alone. Data show the mean F.I. ± SEM of three independent experiments. Statistical significance was calculated by one-way ANOVA. Mean (F.I.) ± SEM values (n = 3); **(F)** ZO-1, T = 1.18 ± 0.11, T SEB = 0.48 ± 0.02, T SEB PS1145 = 1.41 ± 0.27, T SEB S31-201 = 1.19 ± 0.07; CDH1, T = 0.94 ± 0.14, T SEB = 0.58 ± 0.09, T SEB PS1145 = 1.29 ± 0.10, T SEB S31-201 = 1.30 ± 0.17; **(G)** SNAIL-1, T = 0.89 ± 0.45, T SEB = 5.48 ± 0.84, T SEB PS1145 = 1.58 ± 0.69, T SEB S31-201 = 1.13 ± 0.82; TWIST-1, T = 1.09 ± 0.58, T SEB = 4.57 ± 1.06, T SEB PS1145 = 0.46 ± 0.21, T SEB S31-201 = 0.63 ± 0.47; ZEB-1, T = 1.92 ± 0.64, T SEB = 15.49 ± 3.64, T SEB PS1145 = 3.48 ± 2.13, T SEB S31-201 = 2.91 ± 0.86. (*) p < 0.05, (**) p < 0.01, (***) p < 0.001, (****) p < 0.0001. NS, not significant.

Most of the inflammatory cytokines produced by T cells following SEB bivalent binding to the TCR and CD28 exert their functions by activating two main TFs, RelA/NF-κB and STAT3 (23, 35, 55). Consistently, a significant nuclear translocation of RelA/NF-κB (**Figures 4B, C**) and Tyr705 phosphorylated STAT3 (pSTAT3) (56) (**Figures 4D, E**) was observed in Caco-2 cells cultured for 8 h with the inflammatory milieu of SEB-stimulated T cells (**Figure 4A**). Treatment of Caco-2 cells with the NF-κB inhibitor PS1145 (57) or the selective STAT3 inhibitor S31-201 (58) restored both ZO-1 and CDH1 gene expression (**Figure 4F**) and impaired the up-regulation of SNAIL-1, TWIST-1 and ZEB-1 (**Figure 4G**) induced by the inflammatory milieu of SEB-activated T cells.

Altogether, these data highlight a critical role of RelA/NF-κB and STAT3 in inducing epithelial barrier dysfunctions and associated EMT-TFs up-regulation following exposure of Caco-2 cells to SEB-activated inflammatory T cells.

### The inflammatory milieu of SEB-activated T cells promotes the recruitment of pSTAT3 and RelA/NF-κB to the promoters of EMT-TFs in Caco-2 cells

**Figure 5:**
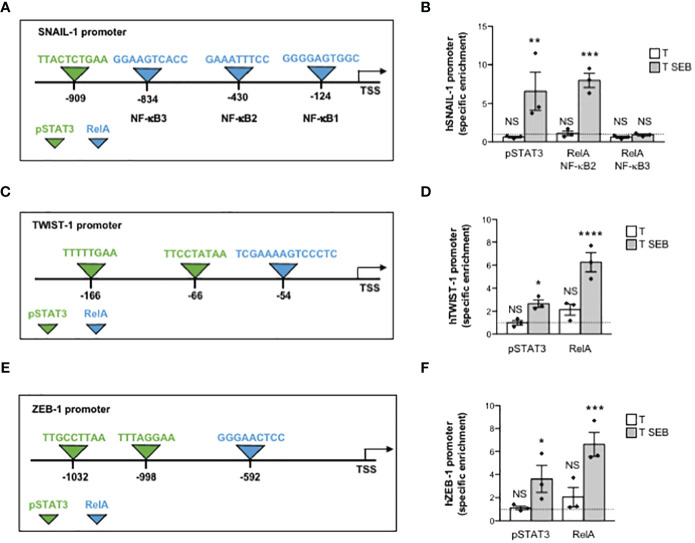
The inflammatory milieu of SEB-stimulated T cells induces the recruitment of RelA/NF-κB and pSTAT3 to the promoters of EMT-TFs. **(A, C, E)** Schematic sequence of the region upstream of the transcriptional starting site (TSS) of human SNAIL-1 **(A)**, TWIST-1 **(C)** and ZEB-1 **(E)** promoter genes. The binding sites of NF-κB (light blue) and STAT3 (yellow) are indicated. **(B, D, F)** Real time PCR for SNAIL-1 **(B)**, TWIST-1 **(D)** and ZEB-1 **(F)** promoters from anti-pSTAT3 and anti-RelA ChIPs performed on Caco-2 cells cultured for 24 h with the culture supernatant (1:4 dilution) of T cells from HD (n = 3) unstimulated (T) or stimulated for 72 h with SEB (T SEB). Specific enrichment over isotype control Abs was calculated by the Cτ method. Data express the mean ± SEM and statistical significance was calculated by one-way ANOVA. Mean ± SEM values (n = 3): **(B)** SNAIL, pSTAT3, T = 0.63 ± 0.10, T SEB = 6.58 ± 2.46; RelA (NF-κB2), T = 1.12 ± 0.30, T SEB = 7.97 ± 0.91; RelA (NF-κB3), T = 0.62 ± 0.14, T SEB = 0.92 ± 0.12; **(D)** TWIST-1, pSTAT3, T = 0.98 ± 0.20, T SEB = 2.66 ± 0.32; RelA, T = 2.14 ± 0.48, T SEB = 6.26 ± 0.83; **(F)** ZEB-1, pSTAT3, T = 1.13 ± 0.15, T SEB = 3.62 ± 1.16 (*) p < 0.05; RelA, T = 2.05 ± 0.83, T SEB = 6.62 ± 1.03. (*) p < 0.05, (**) p < 0.01, (***) p < 0.001, (****) p < 0.0001. NS, not significant.

Several putative binding sites for RelA/NF-κB and STAT3 have been identified within the promoters of SNAIL-1, TWIST-1 and ZEB1 (59–63). In particular, SNAIL-1 promoter contains three putative RelA/NF-κB binding sites, with NF-κB2 and NF-κB3 sites mainly involved in promoting SNAIL-1 expression (63), and one STAT3 binding site (62) near the NF-κB3 site (**Figure 5A**). Two STAT3 (59) and one NF-κB consensus sequence (63) were identified in the promoter of TWIST-1 (**Figure 5C**). Similarly, ZEB-1 promoter contains one NF-κB (61) and two STAT3 binding sites (60) (**Figure 5E**). To assess whether the up-regulation of EMT-TFs induced by the inflammatory milieu of SEB-activated T cells could depend on RelA/NF-κB and/or pSTAT3, we performed chromatin immunoprecipitations (ChIPs) in Caco-2 cells cultured with the supernatants of SEB-activated T cells using specific oligonucleotide probes (**Table 1**). The results evidenced a specific recruitment of both pSTAT3 and RelA on the promoters of SNAIL-1, with RelA that preferentially bound NF-κB2 but not NF-κB3 site (**Figure 5B**). Similarly, selective and specific recruitment of both RelA and pSTAT3 was also observed on TWIST-1 (**Figure 5D**) and ZEB-1 promoters (**Figure 5F**).

Altogether these data evidence that SEB activates T cells to secrete inflammatory cytokines, which in turn promote the activation of RelA/NF-κB and STAT3 and their cooperation in up-regulating EMT-TFs expression.

### A novel pSEB_116-132_ mimetic peptide inhibits inflammatory cytokine production and epithelial barrier dysfunctions induced by SEB-activated T cells

**Figure 6:**
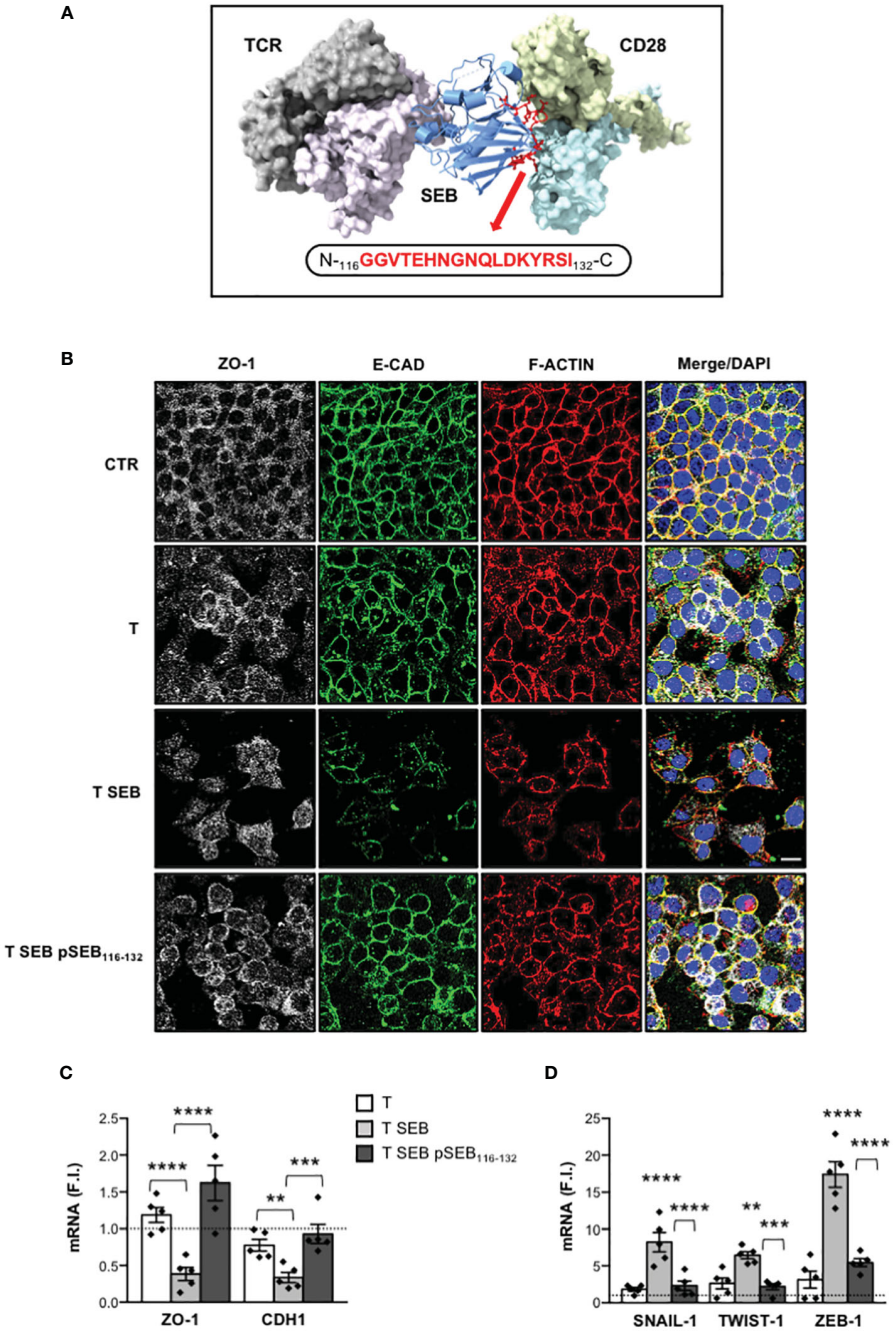
pSEB_116-132_ mimetic peptide targeting the homodimer interface of CD28 restores epithelial barrier integrity and adhesion molecule expression and inhibits EMT-TFs up-regulation. **(A)** Structural model of the TCR–SEB–CD28 complex. The TCR (TCRVβ in light purple and TCRVα in grey) and CD28 dimers (light blue and yellow) are represented as solid surface, SEB in cartoon ribbons (blue). The sequence of pSEB_116-132_ mimetic peptide targeting the region of SEB interacting with the homodimer interface of CD28 is shown in red ball-and-sticks models. **(B)** Caco-2 cells were cultured in 24 trans-well plates for 72 h with medium alone (CTR) or T cells (T), or T cells stimulated with 0.1 μg ml^-1^ SEB (T SEB) in the presence or absence of 10 μM pSEB_116-132_ mimetic peptide. After fixing and permeabilization Caco-2 cells were stained with anti-ZO-1 followed Alexa Fluor 647 Abs (white), anti-E-CAD followed by Alexa Fluor 488 Abs (green), and F-actin was stained with Alexa Fluor 594 phalloidin (red). Nucleus was stained with DPI (blue). Scale bar = 20 μm. **(C, D)** ZO-1 and CDH1 **(C)**, and SNAIL-1, TWIST-1 and ZEB-1 **(D)** mRNA levels in Caco-2 cells cultured for 72 h with medium alone or with T cells from HD (n = 5) or SEB-stimulated T cells (T SEB) in the presence or absence of pSEB_116-132_ mimetic peptide. Values, normalized to GAPDH, were expressed as F.I. over the basal level of Caco-2 cultured with medium alone. Data show the mean F.I. ± SEM and statistical significance was calculated by one-way ANOVA. Mean F.I. ± SEM values (n = 5); **(C)** ZO-1, T = 1.18 ± 0.10, T SEB = 0.38 ± 0.08, T SEB pSEB^116-132 ^= 1.62 ± 0.23; CDH1, T = 0.77 ± 0.08, T SEB = 0.33 ± 0.06, T SEB pSEB^116-132 ^= 0.92 ± 0.12; **(D)** SNAIL.1, T = 1.83 ± 0.25, T SEB = 8.23 ± 1.32, T SEB pSEB^116-132 =^ 2.31 ± 0.63; TWIST-1, T = 2.63 ± 0.75, T SEB = 6.45 ± 0.48, T SEB pSEB^116-132 ^= 2.18 ± 0.40; ZEB-1, T = 3.13 ± 1.15, T SEB = 17.39 ± 1.72, T SEB pSEB^116-132 =^ 5.46 ± 0.53. (**) p < 0.01, (***) p < 0.001, (****) p < 0.0001. NS, not significant.

We have recently demonstrated that SEB may bind the TCR and CD28 in a bivalent manner, thus stimulating the production of inflammatory cytokines independently of APCs (23). Based on the proposed complex between SEB the TCR and CD28, we identified a solvent exposed loop of SEB (residues 116-132) interacting with CD28, and evolutionarily well conserved in other SAgs (**Figure 6A**). We then tested the capability of a mimetic peptide of this region (N-GGVTEHNGNQLDKYRSI-C; hereinafter pSEB_116-132_) to dampen SEB inflammatory activity. Treatment of T cells with pSEB_116-132_ mimetic peptide significantly impaired inflammatory cytokine production induced by SEB (**Supplementary Figure S2**). Moreover, when Caco-2 cells were co-cultured with SEB-stimulated T cells in the presence of pSEB_116-132_ mimetic peptide, F-actin remodelling as well as E-CAD and ZO-1 down-regulation were impaired (**Figure 6B**). Finally, pSEB_116-132_ mimetic peptide also restored ZO-1 and CDH1 gene expression (**Figure 6C**) and inhibited the up-regulation of SNAIL-1, TWIST-1 and ZEB-1 mRNA levels (**Figure 6D**).

Altogether these data disclose a novel pSEB_116-132_ mimetic peptide that by competing with SEB for its binding to the homodimer interface of CD28 is able to dampen inflammatory cytokine production and associated intestinal epithelial cell dysfunctions.

## Discussion

Intestinal epithelial cells are usually exposed to several pro-inflammatory and anti-inflammatory cytokines during chronic inflammation and in inflammatory bowel diseases (IBD) (45). These cytokines are mainly produced by immune cells of the local microenvironment, healing the intestinal mucosa and favouring chronic inflammation, which in some cases may lead to tumour development and progression (52, 64–66). *S. aureus* infections may contribute to intestinal inflammation by secreting SAgs such as SEB that penetrate the epithelial barrier through a transcytosis pathway (33), thus reaching the lamina propria where they stimulate T cells to produce pathogenic inflammatory cytokines (20, 23). Consistently, we found that, by directly binding the TCR and CD28, SEB stimulates T cells to produce inflammatory cytokines such as IFN-γ, IL-6, TNFα, IL-17A and IL-22 (**Figure 1**) (23), which potentially alter the homeostasis and integrity of intestinal epithelial cells (45, 64).

Several inflammatory cytokines may negatively regulate intestinal barrier integrity by modifying the expression and/or localization of cadherins and/or TJs (45). In intestinal epithelial cells, including Caco-2, TNF-α was shown to increase para-cellular permeability by remodelling F-actin cytoskeleton and downregulating both ZO-1 and E-cadherin expressions at both mRNA and protein levels (52, 67–69). The decrease of ZO-1 protein levels induced by TNF-α in Caco-2 cells has been associated to NF-κB-dependent up-regulation of myosin light chain kinase (MLCK) (67, 70) that, by inducing F-actin rearrangement, promotes the dissociation of ZO-1 from TJs ultimately leading to its degradation (52). IFN-γ was found to synergize with TNF-α in inducing TJs disruption and ZO-1 deregulation, favouring both MLCK expression and activation in Caco-2 cells (53, 71, 72). IL-6, a critical pathogenic cytokine in several inflammatory diseases, including IBD (73), enhances intestinal epithelial permeability by impairing TJs integrity and downregulating E-cadherin expression (51, 74, 75). IL-22, a cytokine of the IL-10 family, whose levels increase in colonic tissues and in the peripheral blood of IBD patients (76), was found to increase TJs permeability (54, 77) by downregulating the expression of both ZO-1 and E-cadherin (78). IL-17A was capable to inhibit the expression of E-cadherin in intestinal epithelial cells *in vitro* (79). Interestingly, T lymphocytes stimulated with SEB produced high amounts of TNF-α, IL-6, IFN-γ, IL-22 and IL-17A (23) (**Figures 1A–F**, **Figure 3A**). In Caco-2 cells exposed to the inflammatory milieu of SEB-stimulated T cells, actin cytoskeleton reorganized into actin stress-like fibers (**Figure 1G**) and both ZO-1 and E-cadherin were down-regulated at both protein and mRNA level (**Figure 2**). The neutralization of inflammatory cytokines by NAbs restored Caco-2 cell integrity (**Figure 3**), suggesting that the enterotoxic effects of SEB strongly depend on its T-cell mediated SAg inflammatory activity.

The downregulation of E-CAD and ZO-1 is often associated to the expression of EMT-TFs such as SNAIL, TWIST and ZEB (49, 80, 81). Indeed, the promoter of E-CAD gene (CDH1) contains specific E-box sequences for binding SNAIL-1 (82), TWIST-1 (83) and ZEB-1 (84), which act by repressing CDH1 transcription. Similarly, ZEB-1 acts as a transcriptional repressor of ZO-1 (50, 85, 86). The expression of EMT-TFs is regulated by several signals, including those activated by inflammatory cytokines (80). In this context, Cohen et al. found that T cell-induced inflammatory cytokines mediated the up-regulation of both ZEB-1 and SNAIL-1 in inflammatory breast cancer (87). Goebel et al. showed that activated CD4^+^ T cells induced ZEB-1 expression in a TNF-α- and IL-6-dependent manner, in both pre-malignant and malignant pancreatic ductal epithelial cells (88). IL-17A was found at high levels in the colonic mucosal tissues of patients with Chron’s disease, where it mediated the upregulation of SNAIL-1 while inhibiting E-cadherin expression (79). Similarly, IL-22 has been proven to act on intestinal epithelial cells by inducing the expression of SNAIL-1 and impairing TJs expression and distribution (78). Interestingly, we found a strong up-regulation of SNAIL-1, TWIST-1 and ZEB-1 (**Figure 2F**) in Caco-2 cells co-cultured with SEB-activated T cells, which were inhibited by inflammatory cytokine NAbs (**Figure 3C**). These data suggest an involvement of SEB-induced inflammatory cytokines in the induction of EMT-TFs and the repression of E-Cadherin and ZO-1 expressions. Indeed, many of the inflammatory cytokines secreted by SEB-stimulated T cells signal through NF-κB and STAT3, which are also involved in EMT-TFs expression (89). For example, through the activation and nuclear translocation of pSTAT3 (90), IL-6 up-regulates the expression of SNAIL, TWIST and ZEB in several cancer cell types (91). STAT3 is also activated by IL-22 (92) and IFN-γ (93), suggesting a potential role in amplifying EMT-TFs expression (89). TNF-α (94) and IL-17A (95) mainly activate NF-κB that cooperates with pSTAT3 to induce EMT-TFs expression (50, 80). Consistently with this evidence, the inflammatory milieu of SEB-stimulated T cells (**Figure 4A**) induced a significant nuclear translocation of both RelA/NF-κB (**Figures 4B, C**) and pSTAT3 in Caco-2 cells (**Figures 4D, E**). Treatment of Caco-2 cells with the NF-κB inhibitor PS1145 (57) or the STAT3 inhibitor S31-201 (58, 96) restored ZO-1 and E-cadherin expression (**Figure 4F**) impairing SNAIL-1, TWIST-1 and ZEB-1 up-regulation induced by SEB-stimulated T cells (**Figure 4G**). These data suggest a cooperation of both NF-κB and STAT3 in the transcriptional activation of EMT-TFs promoters.

The promoters of SNAIL-1, TWIST-1 and ZEB-1 contain several putative binding sites for both NF-κB and pSTAT3 (59–63). Notably, by using in silico tools, Pires et al. identified four (-124, -430, -834 and -1119 bp) and six (-54, -249, -870, -956, -983 and -997 bp) putative NF-κB binding sites in the promoter of SNAIL-1 and TWIST-1, respectively (63). A functional consensus sequence for NF-κB binding on ZEB-1 promoter has been also identified by Rajabi et al. at position -592 bp (61). We extended these data by showing that the inflammatory milieu of SEB-activated T cells induced in Caco-2 cells a strong recruitment of RelA/NF-κB on the SNAIL-1 (-430 bp, GGAAATTTCC), TWIST-1 (-54 bp, TCGAAAAGTCCCTC) and ZEB-1 (-592 bp, GGGAACTCC) promoters (**Figure 5**). According to Liu et al. (62), Cheng et al. (59) and Xiong et al. (60), who identified functional pSTAT3 consensus sequences on the promoters of EMT-TFs, we also found that pSTAT3 was recruited to the promoters of SNAIL-1 (-909 bp, TTACTCTGAA), TWIST-1 (-66 bp, TTCCTATAA), and ZEB-1 (-1032 bp, TTGCCTTAA and -998 bp, TTTAGGAA) in Caco-2 cells exposed to SEB-activated T cell secretome (**Figure 5**). Thus, by binding in a bivalent manner the TCR and CD28 on the surface of T cells (23), SEB stimulates the secretion of inflammatory cytokines that in turn mediate the expression of EMT-TFs in a NF-κB- and STAT3-dependent manner.

The inflammatory activity of SEB depends on its capability to stimulate T cells by engaging specific TCRVβ chain elements and the CD28/B7 costimulatory axis either in the presence or absence of MHC-II molecules on APCs (16–22). In particular, extensive studies from Kaempfer and co-workers pointed out a crucial role of CD28 and its co-ligand B7 in binding SEB and in stimulating T cells to produce inflammatory cytokines. Indeed, they identified a 12 amino-acid β-strand (8)/hinge/α-helix (4) conserved region in staphylococcal SAgs that, by engaging the dimer interface of CD28 and B7, enhances their interaction triggering the cytokine storm (16, 18, 19). Furthermore, they also showed that short mimetic peptides targeting the homodimer interface of CD28 were able to attenuate inflammatory cytokine production by interfering with CD28/B7 engagement and signalling induced by SEB (16, 17, 19, 22, 97, 98). More recently, we demonstrated that SEB is able to elicit massive cytokine production by engaging the TCR and CD28 in a bivalent manner and in the absence of APCs expressing B7 molecules (22). By using the cryo-EM structure of SEB-MHCII-TCR-CD3 complex (99) and CD28 (38, 100) together with the previously published spatial-restrained protein-protein docking approach (39, 101, 102), we were able to define that SEB may bind the TCR and CD28 simultaneously by adopting a wedge-like conformation (23). Notably, in our modelling, we identified residues 116-132 of SEB forming a loop that acts as a crucial linker between the two subdomains of SEB (**Figure 6A**). Moreover, this region was mainly responsible for SEB binding to the CD28 homodimer interface. A comparison with the experimentally available structures of other SAgs evidenced that SEB_116-132_ loop is highly exposed and very well conserved from an evolutionary and structural standpoint. Intrigued by these observations, we tested the capability of a short SEB mimetic peptide (pSEB_116-132_) to dampen both inflammatory cytokine production and intestinal epithelial cell dysregulation. Our data demonstrated that pSEB_116-132_ mimetic peptide strongly impaired SEB-mediated inflammatory cytokine production in T cells (**Supplementary Figure S2**) and restored Caco-2 cell integrity (**Figure 6B**) as well as ZO-1 and E-CAD expression (**Figure 6C**) by inhibiting the up-regulation of EMT-TFs (**Figure 6D**).

Overall, our results provide new insights into the enterotoxic activity of SEB during *S. aureus* infections, which may lead to the exacerbation of chronic inflammatory diseases such as IBD (103), especially following infections by MRSA (8, 9, 104), and the identification of a novel mimetic peptide able to attenuate inflammatory-dependent epithelial barrier dysfunctions. *In vivo* experiments to test the efficacy of pSEB_116-132_ mimetic peptide in attenuating or protecting rats or mice from *S. aureus*-induced foodborne intoxication or intestinal inflammation (31, 105, 106) will be seminal to assess its therapeutic potential in *S. aureus*-associated inflammatory diseases.

## Data availability statement

The original contributions presented in the study are included in the article/**Supplementary Material**. Further inquiries can be directed to the corresponding author.

## Ethics statement

The studies involving humans were approved by Ethical Committee of the Policlinico Umberto I (Sapienza University, Rome, Italy). The studies were conducted in accordance with the local legislation and institutional requirements. The participants provided their written informed consent to participate in this study.

## Author contributions

CA: Writing – review & editing, Data curation, Investigation, Methodology. ER: Investigation, Methodology, Writing – review & editing. VT: Methodology, Writing – review & editing. MF: Writing – review & editing, Funding acquisition. AP: Funding acquisition, Writing – review & editing, Conceptualization, Data curation, Methodology, Software. FS: Funding acquisition, Methodology, Writing – review & editing, Investigation. LR: Funding acquisition, Writing – review & editing, Conceptualization. LT: Conceptualization, Funding acquisition, Writing – review & editing, Supervision, Writing – original draft. MK: Conceptualization, Data curation, Investigation, Methodology, Writing – original draft, Writing – review & editing.
