# Supplementary material for: Targeting staphylococcal enterotoxin B binding to CD28 as a new strategy for dampening superantigen-mediated intestinal epithelial barrier dysfunctions
Source: Front Immunol. 2024 Mar 6;15:1365074. doi: 10.3389/fimmu.2024.1365074 (PMC10951378; doi:10.3389/fimmu.2024.1365074)
Supplement: Supplementary file 1 [file DataSheet_1.pdf]

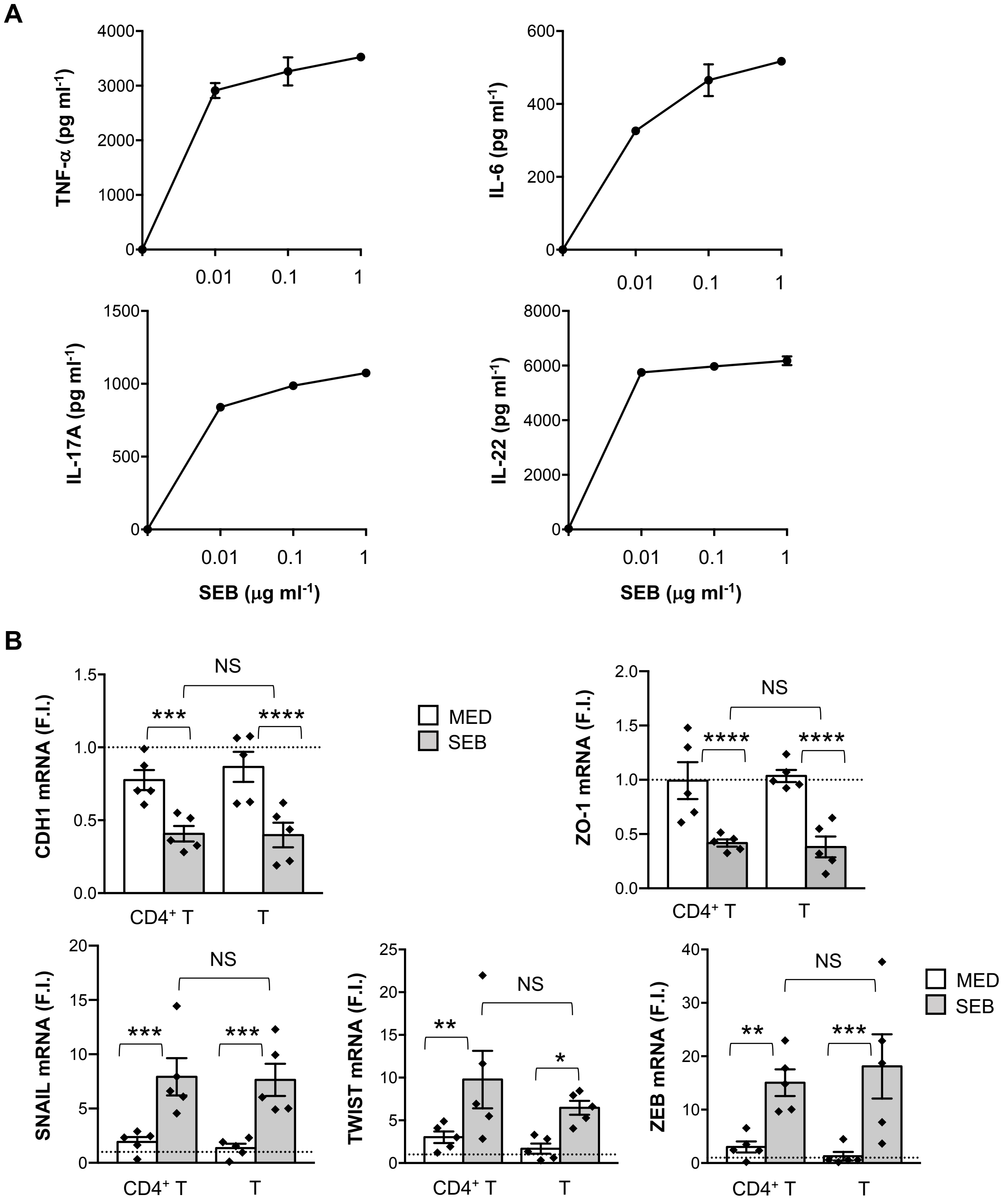

Supplementary Figure S1. (**A**) Cytokine production (ELISA) in culture supernatants of T cells stimulated for 72 h with the indicated doses of SEB. Data show the mean ± SEM of one out of three independent experiments. (**B**) ZO-1, CDH1, SNAIL-1, TWIST-1 and ZEB-1 mRNA levels in Caco-2 cells co-cultured for 72 h with total T cells (T) or CD4+ T cells from HD (n = 5) unstimulated (Med) or stimulated with 0.1 μg ml^{-1} SEB. Values, normalized to GAPDH, were expressed as F.I. over the basal level of Caco-2 cultured with medium alone. Data show the mean F.I. ± SEM and statistical significance was calculated by one-way ANOVA. (*) $p < 0.05$, (**) $p < 0.01$, (***) $p < 0.001$, (****) $p < 0.0001$. NS = not significant.
