# Supplementary material for: Targeting staphylococcal enterotoxin B binding to CD28 as a new strategy for dampening superantigen-mediated intestinal epithelial barrier dysfunctions
Source: Front Immunol. 2024 Mar 6;15:1365074. doi: 10.3389/fimmu.2024.1365074 (PMC10951378; doi:10.3389/fimmu.2024.1365074)
Supplement: Supplementary file 2 [file DataSheet_2.pdf]

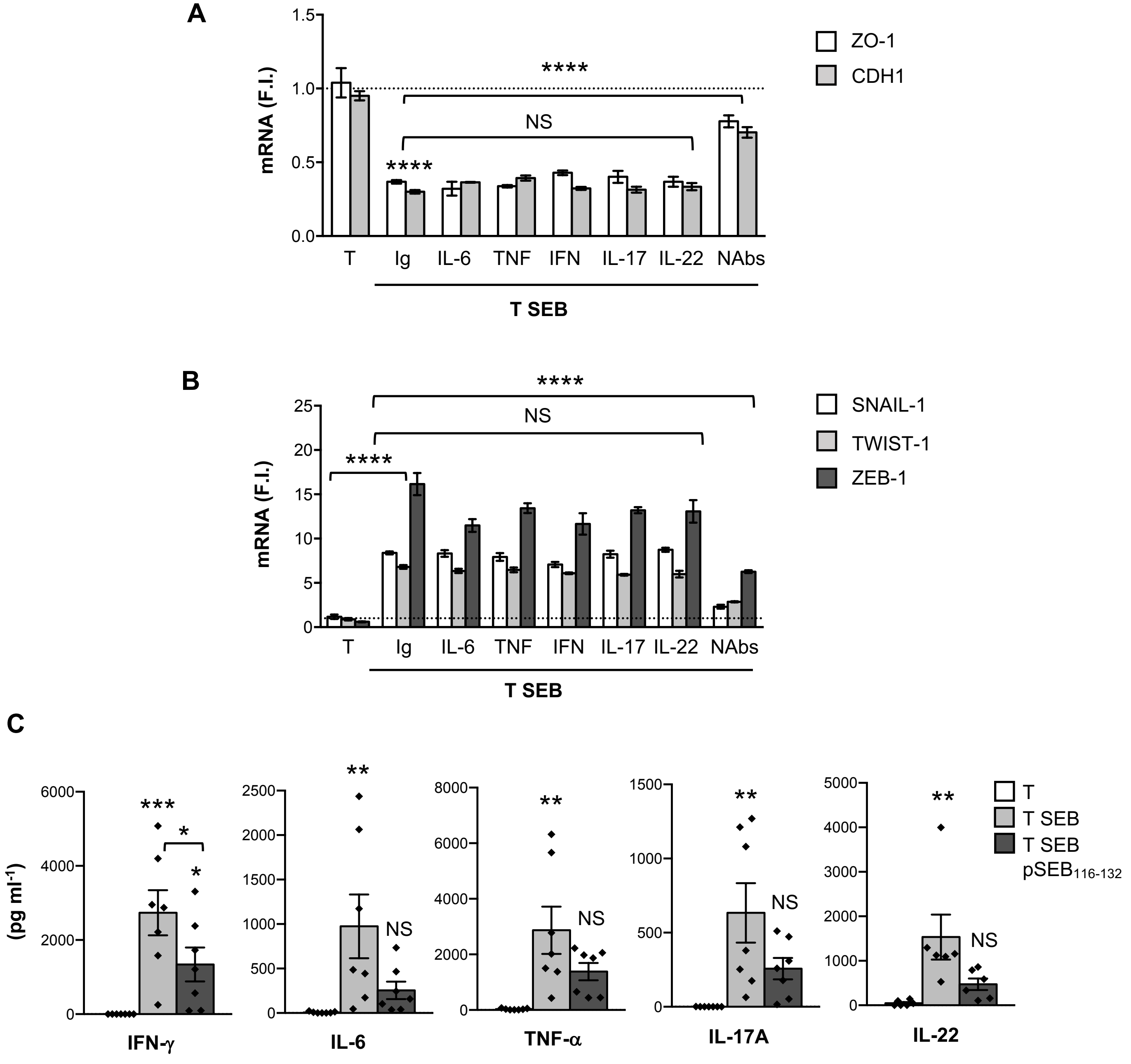

Supplementary Figure S2. (**A, B**) ZO-1 and CDH1 (**A**), and SNAIL-1, TWIST-1 and ZEB-1 (**B**) mRNA levels in Caco-2 cells cultured for 72 h with with T cells or SEB-stimulated T cells (T SEB) in the presence of 2.5 μg ml^{-1} isotype control (Ig) or 2.5 μg ml^{-1} anti-IL-6, anti-TNF-α, anti-IFN-γ, anti-IL-17A, anti-IL-22 neutralising Abs or a combination of all Abs (NAbs). Values, normalized to GAPDH, were expressed as fold inductions (F.I.) over the basal level of Caco-2 cultured with medium alone. Data show the mean F.I. ± SEM and statistical significance was calculated by one-way ANOVA. (**C**) Inflammatory cytokine levels in the supernatants of peripheral T cells from HD (n = 7) unstimulated (T) or stimulated with SEB for 72 h (T SEB) in the presence or absence of 10 μM $pSEB_{116-132}$ mimetic peptide. Data show the mean (pg ml^{-1}) ± SEM and statistical significance was calculated by one-way ANOVA. (*) $p < 0.05$, (**) $p < 0.01$, (***) $p < 0.001$ (****) $p < 0.0001$. NS = not significant
